# Ethyl (2*E*)-2-(hydroxy­imino)propanoate

**DOI:** 10.1107/S1600536810009438

**Published:** 2010-03-20

**Authors:** Igor Vasyl Nikolayenko, Carla Bazzicalupi, Gayle Pamela Thubron, Craig Grimmer

**Affiliations:** a School of Chemistry, University of KwaZulu-Natal, Private Bag X01, Scottsville, Pietermaritzburg, 3209, South Africa; b Department of Chemistry, University of Florence, Via della Lastruccia 3, 50019 Sesto Fiorentino, Florence, Italy

## Abstract

The mol­ecule of the title compound, C_5_H_9_NO_3_, is essentially planar [the maximum deviation for a non-H atom from the mean plane is 0.021 (3) Å] due to the π-conjugation of the hydroxy­imino and carbonyl groups, which are *trans* to each other; *ab initio* calculations *in vacuo* at the DFT (B3LYP/6–311G**++) level of theory confirmed that *E* conformer is indeed the lowest in energy. The packing in crystal structure is influenced by strong inter­molecular O—H⋯N hydrogen-bonding inter­actions between oxime groups and also by π-stacking of the mol­ecules due to the carbonyl and oxime group orbital overlap [inter­planar distance between adjacent mol­ecules = 3.143 (4) Å]. Jointly, these factors afford infinite 6.32 Å thick mol­ecular sheets, where the plane of each mol­ecule is perpendicular to the plane of the sheet. Seen from above, the mol­ecules within the sheet are arranged in a herringbone pattern. Such sheets form a stack due to weak van der Waals inter­actions; the gap between adjacent sheets is 2.07 Å.

## Related literature

The earliest mention of the title compound is probably by Meyer & Züblin (1878 ▶), though the authors ascribed it a nitro­soester structure. It was first prepared in a substantial yield by Ponzio & Ruggeri (1925 ▶). A similar reaction route, based on the condensation of ethyl pyruvate with hydroxyl­amine, was later followed by Jencks (1959 ▶), Armand & Guette (1969 ▶), Pitts *et al.* (2001 ▶) and our group. Jencks (1959 ▶) investigated the kinetics of oxime formation. IR data are presented by Dobrina & Ioffe (1972 ▶) and Ali *et al.* (1988 ▶), while ^1^H-NMR spectra are discussed by Lustig (1961 ▶) and Ali *et al.* (1988 ▶). Quantum mechanical modeling was performed using *JAGUAR* and *MAESTRO* (Schrödinger, 2008 ▶).
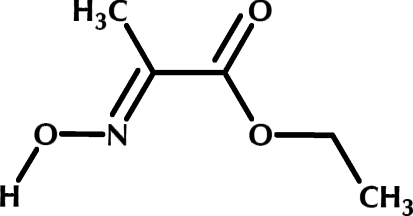


## Experimental

### 

#### Crystal data

C_5_H_9_NO_3_
*M* *_r_* = 131.13
Monoclinic, 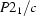

*a* = 11.743 (1) Å
*b* = 4.4227 (6) Å
*c* = 16.860 (2) Å
β = 130.531 (8)°
*V* = 665.55 (14) Å^3^
*Z* = 4
Mo *K*α radiation
μ = 0.11 mm^−1^
*T* = 150 K
0.4 × 0.3 × 0.3 mm

#### Data collection

Oxford Diffraction PX Ultra CCD diffractometer
Absorption correction: multi-scan (*CrysAlis RED*; Oxford Diffraction, 2008 ▶) *T*_min_ = 0.96, *T*_max_ = 0.97
2501 measured reflections
1150 independent reflections
655 reflections with *I* > 2σ(*I*)
*R*_int_ = 0.043

#### Refinement

*R*[*F* ^2^ > 2σ(*F* ^2^)] = 0.049
*wR*(*F* ^2^) = 0.130
*S* = 0.89
1150 reflections
88 parameters
H atoms treated by a mixture of independent and constrained refinement
Δρ_max_ = 0.21 e Å^−3^
Δρ_min_ = −0.20 e Å^−3^

### 

Data collection: *CrysAlis CCD* (Oxford Diffraction, 2008 ▶); cell refinement: *CrysAlis CCD*; data reduction: *CrysAlis RED* (Oxford Diffraction, 2008 ▶); program(s) used to solve structure: *SIR97* (Altomare *et al.*, 1999 ▶); program(s) used to refine structure: *SHELXL97* (Sheldrick, 2008 ▶); molecular graphics: *Mercury* (Macrae *et al.*, 2008 ▶); software used to prepare material for publication: *publCIF* (Westrip, 2010 ▶).

## Supplementary Material

Crystal structure: contains datablocks global, I. DOI: 10.1107/S1600536810009438/bq2193sup1.cif

Structure factors: contains datablocks I. DOI: 10.1107/S1600536810009438/bq2193Isup2.hkl

Additional supplementary materials:  crystallographic information; 3D view; checkCIF report

## Figures and Tables

**Table 1:** Hydrogen-bond geometry (Å, °)

*D*—H⋯*A*	*D*—H	H⋯*A*	*D*⋯*A*	*D*—H⋯*A*
O2—H9⋯N1^i^	0.88 (4)	1.99 (4)	2.778 (3)	148 (3)

Symmetry code: (i) 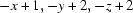.

**Fig. 1.**
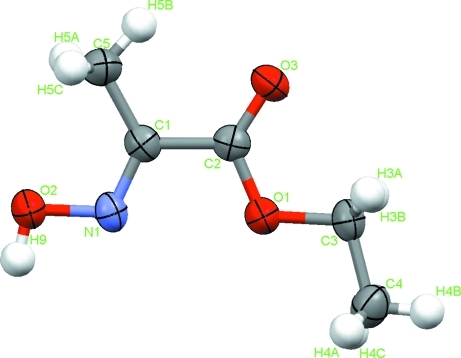
A view of the molecular structure of the title compound. Displacement ellipsoids (Mercury 2.2) are drawn at the 50% probability level.

**Fig. 2.**
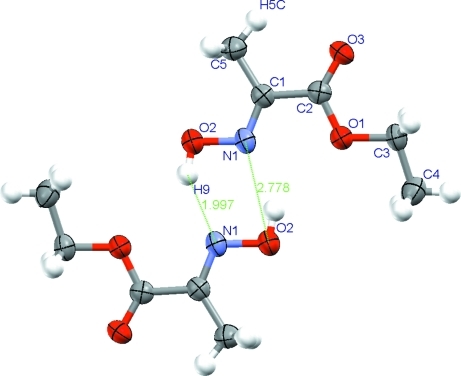
A view of the molecular arrangement of the title compound. Displacement ellipsoids (Mercury 2.2) are drawn at the 50% probability level. Strong O2—H9···N1i and N1···H9i—O2i hydrogen bonding interactions are responsible for the formation of dimers. Symmetry codes: (i) -x+1, -y+2,-z+2.

**Fig. 3.**
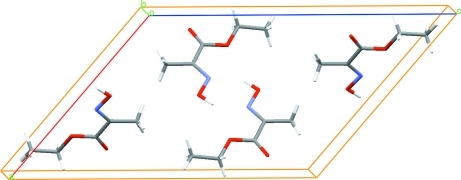
A packing diagram viewed down b-axis for the crystal structure of (I).

**Fig. 4.**
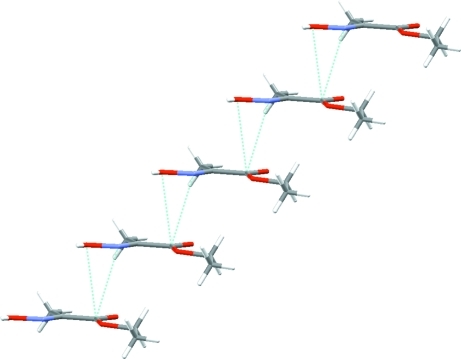
A "staircase" structure induced by π-stacking interactions in (I) as seen from the side.

**Fig. 5.**
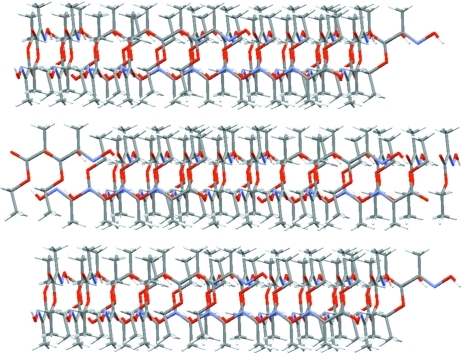
A stack of molecular sheets as seen from the side. The sheets are 6.32 Å thick and are separated by a gap of 2.07 Å.

## supplementary crystallographic information

### Comment

Although the preparation of (I) is well documented (see the Related Literature), no direct structural study has been reported so far. In this communication the molecular and crystal structure of the title compound, determined by a single crystal X-ray diffraction, is presented.

In recent years we became involved in the synthesis and thermodynamic studies of the ligands with chelating oxime-and-amide moieties, as well as their complexes with transition metals. The title compound is the key intermediate for the preparation of such ligands via a condensation route with a suitable diamine (Armand & Guette, 1969).

*Molecular Structure:* The molecule of (I) is profoundly planar (Fig. 1). Maximum deviation for a non-hydrogen atom from the average plane is 0.021 Å. We attribute this to the stabilizing effect of π-conjugation between the hydroxyimino and carbonyl groups. Such interpretation is supported by the *ab initio* quantum mechanical modeling at the DFT (B3LYP/6-311G**++) level of theory (JAGUAR and MAESTRO; Schrödinger, 2008).

In solid state (I) exists as an *E*-isomer, with the oxime and carbonyl groups *trans* to each other. *Ab initio* calculations for (I) in vacuum confirmed that planar *E*-isomer is indeed lower in energy than any of the *Z*-conformers. The only difference of the solid state structure from the lowest energy conformer in vacuum is the orientation of the methyl group riding C1-atom; computed energy of the conformer where H5-atom in plane with the carbonyl group is pointing towards it is 1.71 kJ mol^-1^ lower than for the conformer where such hydrogen atom is pointing away from it. Computationally, planar *E*-conformer is 6.98 kJ mol^-1^ lower in energy than similar *Z*-conformer. When the dihedral angle N1—C1—C2—O3 is varied from 180° to 0°, a potential barrier of 16.6 kJ mol^-1^ is encountered.

Geometric parameters are representative of the hydroxyimino esters. They are in close agreement with the computed ones. For example, the largest difference in the bond length is 0.023 Å (the computed length is longer) for the C8—C5 bond.

*Crystal Structure:* A packing diagram for the crystal structure of (I) is shown in Fig. 3. The spacial arrangement of molecules is influenced by two factors: a) strong intermolecular hydrogen bonding interactions between oxime groups (O2···N1^i^: 2.778 (4) Å, O2···H9—N1^i^: 148.4 °; symmetry code: (i) -x+1, -y+2, -z+2), Fig.2, and b) π-stacking of the molecules due to the carbonyl and oxime group orbital overlap (Fig. 4). The former factor causes the formation of dimers, while the latter one is responsible for a "staircase" structure, where the distance between average planes of adjacent molecules is 3.143 (4) Å. Jointly, these factors afford infinite molecular sheets, where the plane of each individual molecule is perpendicular to the plane of the sheet (Fig. 5). Seen from above, the molecules in the sheet are arranged in a herring-bone pattern. The thickness of such sheets, measured as the distance between two planes drawn through the most external carbon atoms, is 6.32 Å. They form a stack due to weak van der Waals interactions. Measured as above, the gap between adjacent parallel sheets in the stack is 2.07 Å.

### Experimental

Compound (I) was synthesized following a modified procedure of Ponzio & Ruggeri (1925). The reaction between ethylpyruvate and hydroxylamine hydrochloride was carried out at room temperature in aqueous solution. In a typical preparation, hydroxylamine hydrochloride (7.45 g; 105 mmol) was dissolved in 200 ml of water. Sodium carbonate (5.3 g, 50 mmol) was added and the solution stirred for about five minutes. Strong effervescence (evolution of CO_2_) was observed initially. Thereafter ethyl pyruvate (11.3 ml; 100 mmol) was added drop-wise and the solution was left to stir for half an hour.

After about 20 min, large quantity of a flaky white precipitate was observed. The precipitate was subsequently filtered off, rinsed with cold water, and dried on a watch glass. Remaining in aqueous layer (I) was extracted with dichloromethane (2×100 ml). The organic fractions were combined, dried over magnesium sulphate, and the solvent removed. The solid recovered was combined with the primary precipitate. This crude product was recystallised from hot ethanol, affording nearly quantitative yield (typical figures: 95-98 %).

Colorless silky crystals in the shape of elongated prisms were characterized by the melting point determination, FTIR, NMR, GCMS, MS/ToF, and X-ray diffraction.

Melting point temperature. Stanford Research Systems MPA 100 Optmelt.

95.6–96.7 °C.

FTIR. Perkin-Elmer Spectrum One.

(KBr, cm^-1^): 732, 753 (N–O), 782, 854, 974, 1019 (C–O–C), 1117, 1179 (O–H), 1313, 1368, 1390, 1447, 1469, 1716 ν(C=N), 1726 ν(C=O), 2875, 2910, 2981, 3008, ν(C–H, CH_2_, CH_3_), 3243 ν(O–H).

NMR. Varian Unity Inova 500, Oxford magnet 11.744 T.

^1^H NMR (CDCl_3_, 499.98 MHz), δ: 1.341 (t, 3H, *J *= 7.15 Hz, CH_3_, C1), 2.097 (s, 3H, CH_3_, C5), 4.314 (q, 2H, *J *= 7.15 Hz, CH_2_, C2), ca 9.5 (s, br, 1H, OH).

^13^C NMR (CDCl_3_, 125.736 MHz), δ: 10. 453 (CH_3_, C5), 14.027 (CH_3_, C1), 61.817 (CH_2_, C2), 149.425 (C4), 163.699 (C3).

^1^H NMR (DMSO-d_6_, 499.98 MHz), δ: 1.232 (t, 3H, *J *= 7.15 Hz, CH_3_, C1), 1.918 (s, 3H, CH_3_, C5), 4.184 (q, 2H, *J* = 7.15 Hz, CH_2_, C2), 12.203 (s, 1H, OH).

^13^C NMR (DMSO-d_6_, 125.736 MHz), δ: 10. 494 (CH_3_, C5), 14.020 (CH_3_, C1), 60.766 (CH_2_, C2), 147.768 (C4), 163.994 (C3).

GCMS. ThermoFinnigan Trace GC - PolarisQ MS

MS [CI]: *m/z* (%) 58.0 (86 %), 86.0 (100 %), 104.0 (73 %), 132.1 (66 %) [M]^+^

MS/ToF. Waters Micromass LCT Premier.

MS [ES+]: *m/z* (%) Calculated for [C_5_H_9_NO_3_Na]^+^ 154.0480; found 154.0474 (100%); δ -3.9 ppm

The melting point range is reported from the onset point to the clear point. It was determined at a heating rate of 1 °C min^-1^ with the apparatus calibrated against melting points of vanillin, phenacetin, and caffeine SRS melting point standards, traceable to the WHO standards.

Assignment of chemical shifts in the NMR-spectra is based on the analysis of one-dimensional (^1^H, ^13^C, dept) and correlation two-dimensional (gCOSY, ghmqc, ghsqc) spectra.

Fragmentation in the GCMS spectrum is mainly due to the McLafferty rearrangement of (I); the masses of expected fragments are: 28, 58, 73, 85, and 103.

### Refinement

All H atoms were positioned geometrically and allowed to ride on their parent atoms, with C—H = 0.93–0.98 Å and *U*_iso_(H) = 1.2–1.5 *U*_eq_(C).

### Crystal data

**Table:**

C_5_H_9_NO_3_	*F*(000) = 280
*M**_r_* = 131.13	*D*_x_ = 1.309 Mg m^−^^3^
Monoclinic, *P*2_1_/*c*	Melting point: 369.0 K
Hall symbol: -p 2ybc	Mo *K*α radiation, λ = 0.71073 Å
*a* = 11.743 (1) Å	Cell parameters from 842 reflections
*b* = 4.4227 (6) Å	θ = 3.9–27.2°
*c* = 16.860 (2) Å	µ = 0.11 mm^−^^1^
β = 130.531 (8)°	*T* = 150 K
*V* = 665.55 (14) Å^3^	Prism, colorless
*Z* = 4	0.4 × 0.3 × 0.3 mm

### Data collection

**Table:**

Oxford Diffraction PX Ultra CCD diffractometer	1150 independent reflections
Radiation source: Fine-focus sealed tube	655 reflections with *I* > 2σ(*I*)
graphite	*R*_int_ = 0.043
Detector resolution: 16.4547 pixels mm^-1^	θ_max_ = 25.0°, θ_min_ = 4.6°
ω scans	*h* = −13→13
Absorption correction: multi-scan (*CrysAlis RED*; Oxford Diffraction, 2008)	*k* = −5→5
*T*_min_ = 0.96, *T*_max_ = 0.97	*l* = −19→15
2501 measured reflections	

### Refinement

**Table:**

Refinement on *F*^2^	Primary atom site location: structure-invariant direct methods
Least-squares matrix: full	Secondary atom site location: difference Fourier map
*R*[*F*^2^ > 2σ(*F*^2^)] = 0.049	Hydrogen site location: inferred from neighbouring sites
*wR*(*F*^2^) = 0.130	H atoms treated by a mixture of independent and constrained refinement
*S* = 0.89	*w* = 1/[σ^2^(*F*_o_^2^) + (0.0705*P*)^2^ + ] where *P* = (*F*_o_^2^ + 2*F*_c_^2^)/3
1150 reflections	(Δ/σ)_max_ < 0.001
88 parameters	Δρ_max_ = 0.21 e Å^−^^3^
0 restraints	Δρ_min_ = −0.20 e Å^−^^3^

### Special details

**Table:**

Experimental. (CrysAlis RED; Oxford Diffraction, 2008) Empirical absorption correction using spherical harmonics, implemented in SCALE3 ABSPACK scaling algorithm.
Geometry. All esds (except the esd in the dihedral angle between two l.s. planes) are estimated using the full covariance matrix. The cell esds are taken into account individually in the estimation of esds in distances, angles and torsion angles; correlations between esds in cell parameters are only used when they are defined by crystal symmetry. An approximate (isotropic) treatment of cell esds is used for estimating esds involving l.s. planes.
Refinement. Refinement of *F*^2^ against ALL reflections. The weighted *R*-factor wR and goodness of fit *S* are based on *F*^2^, conventional *R*-factors are based on *F*, with *F* set to zero for negative *F*^2^. The threshold expression of *F*^2^ > σ(*F*^2^) is used only for calculating *R*-factors(gt) etc., and is not relevant to the choice of reflections for refinement. *R*-factors based on *F*^2^ are statistically about twice as large as those based on *F*, and *R*- factors based on ALL data will be even larger.

### Fractional atomic coordinates and isotropic or equivalent isotropic displacement parameters (Å^2^)

**Table:**

	*x*	*y*	*z*	*U*_iso_*/*U*_eq_	
O1	0.2376 (2)	0.4841 (4)	0.88980 (14)	0.0406 (6)	
O2	0.5119 (2)	1.0534 (5)	0.90703 (16)	0.0443 (6)	
O3	0.1496 (2)	0.3150 (4)	0.73304 (15)	0.0449 (6)	
N1	0.4183 (2)	0.8478 (5)	0.90293 (17)	0.0376 (6)	
C1	0.3385 (3)	0.6943 (6)	0.8184 (2)	0.0363 (7)	
C2	0.2319 (3)	0.4760 (6)	0.8080 (2)	0.0356 (7)	
C3	0.1312 (3)	0.2900 (7)	0.8827 (2)	0.0430 (8)	
H3A	0.1534	0.0792	0.8822	0.052*	
H3B	0.0301	0.3321	0.8190	0.052*	
C4	0.1452 (4)	0.3545 (7)	0.9759 (2)	0.0552 (9)	
H4A	0.1230	0.5637	0.9756	0.083*	
H4B	0.2455	0.3111	1.0385	0.083*	
H4C	0.0759	0.2301	0.9735	0.083*	
C5	0.3381 (4)	0.7196 (7)	0.7309 (2)	0.0516 (9)	
H5A	0.4368	0.6785	0.7556	0.077*	
H5B	0.3087	0.9204	0.7026	0.077*	
H5C	0.2686	0.5762	0.6776	0.077*	
H9	0.561 (4)	1.124 (8)	0.970 (3)	0.094 (14)*	

### Atomic displacement parameters (Å^2^)

**Table:**

	*U*^11^	*U*^22^	*U*^33^	*U*^12^	*U*^13^	*U*^23^
O1	0.0409 (13)	0.0400 (12)	0.0408 (12)	−0.0054 (10)	0.0265 (11)	−0.0035 (9)
O2	0.0432 (13)	0.0419 (13)	0.0468 (14)	−0.0088 (10)	0.0288 (12)	−0.0042 (10)
O3	0.0432 (13)	0.0421 (13)	0.0419 (11)	−0.0071 (10)	0.0243 (11)	−0.0105 (10)
N1	0.0323 (14)	0.0324 (14)	0.0442 (15)	0.0003 (12)	0.0231 (13)	0.0000 (12)
C1	0.0344 (17)	0.0318 (16)	0.0386 (16)	0.0040 (14)	0.0218 (15)	0.0017 (14)
C2	0.0345 (17)	0.0324 (16)	0.0370 (17)	0.0072 (15)	0.0219 (15)	0.0034 (14)
C3	0.0412 (19)	0.0355 (17)	0.0501 (17)	−0.0035 (14)	0.0288 (16)	0.0007 (14)
C4	0.062 (2)	0.055 (2)	0.067 (2)	0.0007 (18)	0.050 (2)	0.0024 (17)
C5	0.061 (2)	0.052 (2)	0.0507 (18)	−0.0053 (17)	0.0401 (18)	−0.0066 (16)

### Geometric parameters (Å, °)

**Table:**

O1—C2	1.338 (3)	C3—H3A	0.9700
O1—C3	1.456 (3)	C3—H3B	0.9700
O2—N1	1.393 (3)	C4—H4A	0.9600
O2—H9	0.88 (4)	C4—H4B	0.9600
O3—C2	1.204 (3)	C4—H4C	0.9600
N1—C1	1.279 (3)	C5—H5A	0.9600
C1—C5	1.476 (4)	C5—H5B	0.9600
C1—C2	1.498 (4)	C5—H5C	0.9600
C3—C4	1.498 (4)		
			
C2—O1—C3	115.8 (2)	H3A—C3—H3B	108.5
N1—O2—H9	100 (2)	C3—C4—H4A	109.5
C1—N1—O2	112.6 (2)	C3—C4—H4B	109.5
N1—C1—C5	126.7 (3)	H4A—C4—H4B	109.5
N1—C1—C2	115.1 (2)	C3—C4—H4C	109.5
C5—C1—C2	118.2 (3)	H4A—C4—H4C	109.5
O3—C2—O1	124.5 (3)	H4B—C4—H4C	109.5
O3—C2—C1	122.9 (2)	C1—C5—H5A	109.5
O1—C2—C1	112.6 (3)	C1—C5—H5B	109.5
O1—C3—C4	107.4 (2)	H5A—C5—H5B	109.5
O1—C3—H3A	110.2	C1—C5—H5C	109.5
C4—C3—H3A	110.2	H5A—C5—H5C	109.5
O1—C3—H3B	110.2	H5B—C5—H5C	109.5
C4—C3—H3B	110.2		
			
O2—N1—C1—C2	−178.2 (2)	N1—C1—C2—O1	1.0 (3)
N1—C1—C2—O3	−179.8 (3)		

### Hydrogen-bond geometry (Å, °)

**Table:**

*D*—H···*A*	*D*—H	H···*A*	*D*···*A*	*D*—H···*A*
O2—H9···N1^i^	0.88 (4)	1.99 (4)	2.778 (3)	148 (3)

Symmetry codes: (i) −*x*+1, −*y*+2, −*z*+2.
